# *N*-methyl-d-aspartate receptors induce M1 polarization of macrophages: Feasibility of targeted imaging in inflammatory response in vivo

**DOI:** 10.1186/s13578-023-01007-5

**Published:** 2023-03-30

**Authors:** Hui-Jeon Jeon, Jun-Kyu Byun, Sang Bong Lee, Kwang Hee Son, Ji-Youn Lim, Da Sol Lee, Kil Soo Kim, Jin Woo Park, Gyeong Rim Shin, Ye Jin Kim, Jonghwa Jin, Daehoon Kim, Dong-Ho Kim, Ji Hoon Yu, Yeon-Kyung Choi, Keun-Gyu Park, Yong Hyun Jeon

**Affiliations:** 1 New Drug Development Center, Daegu-Gyeongbuk Medical Innovation Foundation (KMEDIhub), 80 Chembok-Ro, Daegu, 41061 South Korea; 2 BK21 FOUR Community-Based Intelligent Novel Drug Discovery Education Unit, Research Institute of Pharmaceutical Sciences, College of Pharmacy, Kyungpook National University, Daegu, 41566 South Korea; 3 THERABEST, Co., Ltd, 06657 Seoul, South Korea; 4 Preclinical Research Center, Daegu-Gyeongbuk Medical Innovation Foundation (KMEDIhub), 41061 Daegu, South Korea; 5 College of Veterinary Medicine, Kyungpook National University, Daegu, 41566 South Korea; 6 BioActs.Co., Ltd., 21666 Incheon, South Korea; 7 Department of Internal Medicine, School of Medicine, Kyungpook National University, Kyungpook National University Hospital, Daegu, 41944 South Korea; 8 Research Institute of Aging and Metabolism, Kyungpook National University, Daegu, 41566 South Korea; 9 Department of Biomedical Science, Kyungpook National University, Daegu, 41566 South Korea; 10 Department of Internal Medicine, School of Medicine, Kyungpook National University, Kyungpook National University Chilgok Hospital, Daegu, 41404 South Korea; 11 Leading-Edge Research Center for Drug Discovery and Development for Diabetes and Metabolic Disease, Kyungpook National University Hospital, Daegu, 41404 South Korea

**Keywords:** Macrophage, *N*-methyl-d-aspartate receptors, Inflammation, Antibody-mediated imaging, Near-infrared fluorescent

## Abstract

**Background:**

*N*-methyl-d-aspartate receptors (NMDARs) are considered to be involved in several physiological and pathophysiological processes in addition to the progression of neurological disorders. However, how NMDARs are involved in the glycolytic phenotype of M1 macrophage polarization and the possibility of using them as a bio-imaging probe for macrophage-mediated inflammation remain unclear.

**Methods:**

We analyzed cellular responses to NMDAR antagonism and small interfering RNAs using mouse bone marrow-derived macrophages (BMDMs) treated with lipopolysaccharide (LPS). An NMDAR targeting imaging probe, N-TIP, was produced via the introduction of NMDAR antibody and the infrared fluorescent dye FSD Fluor™ 647. N-TIP binding efficiency was tested in intact and LPS-stimulated BMDMs. N-TIP was intravenously administered to mice with carrageenan (CG)- and LPS-induced paw edema, and in vivo fluorescence imaging was conducted. The anti-inflammatory effects of dexamethasone were evaluated using the N-TIP-mediated macrophage imaging technique.

**Results:**

NMDARs were overexpressed in LPS-treated macrophages, subsequently inducing M1 macrophage polarization. Mechanistically, NMDAR-mediated Ca^2+^ accumulation resulted in LPS-stimulated glycolysis via upregulation of PI3K/AKT/mTORC1 signaling. In vivo fluorescence imaging with N-TIP showed LPS- and CG-induced inflamed lesions at 5 h post-inflammation, and the inflamed lesions could be detected until 24 h. Furthermore, our N-TIP-mediated macrophage imaging technique helped successfully visualize the anti-inflammatory effects of dexamethasone in mice with inflammation.

**Conclusion:**

This study demonstrates that NMDAR-mediated glycolysis plays a critical role in M1 macrophage-related inflammation. Moreover, our results suggest that NMDAR targeting imaging probe may be useful in research on inflammatory response in vivo.

**Supplementary Information:**

The online version contains supplementary material available at 10.1186/s13578-023-01007-5.

## Background

Macrophages are critical in a broad range of host defense and immunological processes [1]. In response to different local microenvironments, resting macrophages polarize into M1 (pro-inflammatory) or M2 (anti-inflammatory) macrophage [2]. M1 macrophages are activated by lipopolysaccharide (LPS) during infection or tissue injury, and they produce a wide range of pro-inflammatory cytokines and immune responses. Molecular biological methods have provided critical information on macrophage biology. However, the in vivo dynamic behaviors of macrophages remain poorly understood. Therefore, a highly specific probe for activated M1 macrophages is required to better understand macrophage function and dynamics as well as responses to therapeutic interventions under inflammatory conditions.

M1 macrophages reprogram their metabolism toward enhanced glycolysis to facilitate increased cytokine production in response to inflammatory signals [3–5]. The phosphoinositide 3-kinase (PI3K)/threonine protein kinase B (PKB; also known as AKT)/mechanistic target of rapamycin complex 1 (mTORC1) pathway enhances glucose uptake and aerobic glycolysis to support the production of pro-inflammatory cytokines, including TNF-**α** and IL-6 [6]. mTORC1 further integrates diverse metabolic signals for M1 activation by increasing lipogenesis and protein synthesis, which can modulate the production of high levels of chemokines, cytokines, and other highly induced factors in activated M1 macrophages [7]. Thus, it is crucial to find a novel target mediating mTORC1-induced glycolysis and M1 polarization in macrophages to facilitate the development of new imaging techniques.

*N*-methyl-d-aspartate receptors (NMDARs) are ionotropic glutamate receptors known for governing synaptic plasticity in the central nervous system. They play an important role in learning, memory, and neuron maturation [8]. Activation of NMDARs by binding of two distinct agonists, glutamate and glycine, allows permeability of Ca^2+^, Na^+^, and K^+^; among them, Ca^2+^ signaling accounts for most of the NMDAR-mediated functions [9]. Functional NMDARs are composed of two obligatory NR1/GluN1 subunits and two regulatory subunits from either the NR2 or GluN2 subunit [10]. NMDARs are known to influence synaptic plasticity and long-term potentiation, which is critical for learning and memory processes. Abnormal NMDAR functioning is associated with neurological diseases, including schizophrenia, cognitive disabilities, and Parkinson’s disease [11]. There is also mounting evidence on the detection of NMDARs in various types of human cancers and macrophages, attracting attention to the functional significance of NMDARs in various implicated diseases [12, 13]. The NMDAR receptor-mediated calcium signaling pathway generates reactive oxygen species (ROS), which lead to enhanced COX-2 expression in macrophages [14]. In addition, ketamine (an NMDAR antagonist)-induced anti-inflammatory M2 profiles support the sustained beneficial effects of ketamine in patients with major depressive disorder [15]. However, it is still largely unknown how NMDARs play a role in M1 macrophage polarization and whether they can be applied to monitor inflammatory response in various inflammatory diseases.

Here, we aimed to elucidate the role of NMDARs in metabolic reprogramming required for M1 polarization and the possibility of visualization of inflammatory lesions in mice, using a combination of antibody-based optical imaging and infrared fluorescence (FL)-labeling techniques.

## Materials and methods

### Cells

Mouse bone marrow derived macrophages (BMDMs) were collected from the tibia and femur of pathogen-free six-week-old female C57BL/6 mice obtained from DooYeol Biotech. (Seoul, South Korea). The cells were cultured in α-Minimal Essential Medium containing 30% L929 cell-conditioned medium and 10% FBS for 7 days. The medium was changed every 2–3 days.

### Ethics statement

All animals were maintained according to the Guidelines for the Care and Use of Laboratory Animals of the Institute of Laboratory Animal Center, Daegu-Gyeongbuk Medical Innovation Foundation (Approval Number: DGMIF-17061301-00).

### Materials

All chemical reagents including hexadecyl trimethyl ammonium bromide (CTAB), tetraethyl orthosilicate, 3-mercaptopropyl-triethoxysilane (MPTES), lipopolysaccharides (LPS) from *Escherichia coli* O111:B4, MK-801, glutamate, NMDA, rapamycin, BAPTA-AM, N-(6-aminohexyl)-5-chloro-1-naphthalenesulfonamide (W-7), and trifluoperazine (TFP) were purchased from Sigma Aldrich Chemical Co. (St. Louis, MO, USA). Rabbit IgG isotype control was obtained from Abcam (Cambridge, United Kingdom). NMDAR1 polyclonal antibody was obtained from Invitrogen of Thermo Fisher Scientific Co. (Waltham, MA, USA). CellMask™ green plasma membrane stain and Hoechst 33342 (Trihydrochloride) were purchased from Thermo Fisher Scientific. Phosphate buffered saline (PBS, pH7.4) and 4% paraformaldehyde (PFA) were supplied by Biosesang (Seoungnam, South Korea). FSD Fluor™ 647 dye was purchased from BioActs Co. (Incheon, South Korea).

### Chemical treatment

Cells were treated with the NMDAR antagonist MK-801 (150 μM), LPS (100 ng/mL), the mTOR inhibitor rapamycin (25 nM), the calcium chelator BAPTA-AM (2 μM), glutamate (300 μM), NMDA (300 μM), and the calmodulin inhibitor W-7 (20 μM) or TFP (20 μM) for 24 h.

### siRNA transfection

BMDMs were transfected with control siRNA, mouse siGrin1, sip65 (Santa Cruz biotechnology, Dallas, TX, USA) using VIROMER Blue (Lipocalyx, Weinbergweg, Halle, Germany).

### Western blot analysis

Protein from BMDMs lysates was resolved NuPAGE 4%-12% gels (Thermo Fisher Scientific) or Tris–Glycine gel, and transferred to PVDF membranes (Millipore, Burlington, MA, USA). After blocking with 5% skim milk, the membranes were incubated with primary antibodies at 4 °C overnight. Primary antibodies against the following proteins: iNOS, HIF-1α (Novus, Centennial, CO, USA), NMDAR1 (Abcam), p-p65 (S536), p65, p-PI3K (Y458), PI3K, p-AKT (T308), AKT, p-p70S6K (T389), p70S6K, and LDHA (Cell Signaling Technology, Danvers, MA, USA), and Actin (Sigma). The HRP-conjugated secondary antibodies were used to incubate the membranes for 2 h at room temperature. Membranes were detected using Image Quant LAS-4000 (GE Healthcare, Chicago, IL, USA).

### Quantitative RT-PCR

The total RNA from BMDMs were isolated using the Trizol Reagent (Thermo Fisher Scientific) and total RNA was reverse transcribed into cDNA using the RevertAid First Strand cDNA Synthesis Kit (Thermo Fisher Scientific). cDNA was used for subsequent qRT-PCR using the SYBR-Green PCR Master Mix (Applied Biosystems, Foster City, CA, USA). The primer sequences were as follows: Grin1 forward, AACAGCAACAAAAAGGAGTGGAA, and reverse, GCGCACGCTCATTGTTAATG; Grin2A forward, GAACGCGAACTTCGAAATCTG, and reverse, TCTTAGGGTCAGTGCGGTTCA; Grin2B forward, CTGCCCTCCTCCAAACACA, and reverse, CATCCGATCGAATCAAGTCA; 36B4 forward, ACCTCCTTCTTCCAGGCTTT, and reverse, CTCCAGTCTTTATCAGCTGC. Gene expression was normalized to that of the endogenous reference gene, 36B4.

### Measurement of cytokines

The concentrations of cytokines were measured using mouse IL-1β, TNF-α, and IL-6 ELISA kit purchased from Thermo Fisher Scientific. Data were normalized against standard controls in the kit.

### ECAR measurement

Cells were seeded into the XF24 Microplates (Agilent, Santa Clara, CA, USA) to measure the extracellular acidification rate (ECAR: an indicator of glycolysis) using seahorse XF24 analyzer (Agilent). ECAR readings were measured from each treatment groups. Seahorse datasets were normalized to the protein contents.

### Measurement of calcium levels

BMDMS were seeded overnight at 5 × 10^4^ cells per well in a 96-well black wall/clear bottom microplate. Cytosolic Ca^2+^ levels in BMDMs were measured using FLUOFORTE^®^ Calcium assay kit (Enzo Life Sciences, Farmingdale, NY, USA).

### Production of NMDAR-FSD Fluor™ 647 (N-TIP) and Isotype-FSD Fluor.™ 647 (I-TIP)

2 mg/mL solution of Rabbit IgG monoclonal Isotype Control (Abcam, Prod # ab172730) and Anti-NMDAR1 antibody (Thermo Fisher Scientific, Prod # PA3-102) in pH 7.4 PBS buffer at ambient temperature was treated with 20-fold excess FSD Fluor™ 647 (BioActs, Prod # KOSC1315) and shaken for 1 h in a dark. Reaction mixtures were loaded in Sephadex G-25 columns, purified by eluting with pH 7.4 PBS buffer. Then dye-conjugated antibodies were concentrated more than 1 mg/mL. To analyze the optical properties of both conjugates, absorbance spectra were obtained with Nanodrop 2000 (Thermo Fisher Scientific), and fluorescence spectra were analyzed with LS 55 Fluorescence Spectrometer (PerkinElmer) (Additional file 1: Fig. S1). NMDAR-FSD Fluor™ 647 was briefly called as N-TIP (NMDAR Targeting Imaging Probe), and Isotype-FSD Fluor™ 647 was called as I-TIP (IgG Targeting Imaging Probe).

$$\mathbf{F}\mathbf{l}\mathbf{u}\mathbf{o}\mathbf{r}\mathbf{o}\mathbf{p}\mathbf{h}\mathbf{o}\mathbf{r}\mathbf{e}\mathbf{t}\mathbf{o}\,\mathbf{P}\mathbf{r}\mathbf{o}\mathbf{t}\mathbf{e}\mathbf{i}\mathbf{n}\,\mathbf{R}\mathbf{a}\mathbf{t}\mathbf{i}\mathbf{o}\,\left(\mathbf{F}/\mathbf{P}\mathbf{R}\mathbf{a}\mathbf{t}\mathbf{i}\mathbf{o}\right)=\frac{{{\varvec{\varepsilon}}}_{\boldsymbol{ }{\varvec{p}}{\varvec{r}}{\varvec{o}}{\varvec{t}}{\varvec{e}}{\varvec{i}}{\varvec{n}}}\times {{\varvec{A}}}_{{\varvec{m}}{\varvec{a}}{\varvec{x}}}}{{{\varvec{\varepsilon}}}_{\boldsymbol{ }{\varvec{d}}{\varvec{y}}{\varvec{e}}}\left\{{{\varvec{A}}}_{280}-\left({{\varvec{A}}}_{{\varvec{m}}{\varvec{a}}{\varvec{x}}}\times {{\varvec{C}}{\varvec{F}}}_{280}\right)\right\}}$$

${{\varvec{\varepsilon}}}_{\boldsymbol{ }{\varvec{p}}{\varvec{r}}{\varvec{o}}{\varvec{t}}{\varvec{e}}{\varvec{i}}{\varvec{n}}}$ = Protein molar extinction coefficient. The typical molar extinction coefficient of IgGantibody is ~ 210,000 M^-1^cm^-1^${{\varvec{\varepsilon}}}_{\boldsymbol{ }{\varvec{d}}{\varvec{y}}{\varvec{e}}}$ = molar extinction coefficient of FSD Fluor™ 647 (239,000 M^−1^ cm.^−1^)

${{\varvec{A}}}_{{\varvec{m}}{\varvec{a}}{\varvec{x}}}$ = Absorbance of Protein–dye conjugate at ${\uplambda }_{max}$(nm) for dye.

${{\varvec{A}}}_{280}$ = Absorbance of Protein–dye conjugate at ${\uplambda }_{max}$(nm) for protein.

${{\varvec{C}}{\varvec{F}}}_{280}$ = Correction factor; adjusts for the amount of absorbance at 280 nm caused by the dye; 0.03 for FSD Fluor™ 647.

### Flow cytometry

BMDMs were processed into single cell suspensions, then fixated 4% paraformaldehyde for 10 min at 4 °C, and incubated with antibodies (Mouse F4/80-FITC, and Mouse CD11b-FITC; Thermo Fisher Scientific, I-TIP, N-TIP) for 1 h at 4 °C. The cells were then washed twice with flow buffer, and resuspended in 0.5 ml of flow buffer for analysis. Flow cytometry was performed using a FACSCalibur flow cytometer (BD Biosciences, San Jose, CA, USA). Flow cytometric analysis was performed on FlowJo software (FlowJo, Ashland, OR, USA).

### Isolation of peritoneal macrophages

Eight-week-old male *C57BL/6 J* mice were intraperitoneally (i.p.) injected with LPS (2 mg/kg). At 10 min post-injection, the mice were i.p. injected with N-TIP (0.4 mg/kg) or I-TIP (0.4 mg/kg) for 24 h. Peritoneal cells were harvested through peritoneal lavage and then western blot analysis was performed.

### Three-dimensional (3D)-based image analysis

BMDMs were stabilized after seeding in a 96-well plate, a black wall surface on a transparent bottom. Cells were treated with or without LPS for 24 h. Prior to the imaging, the cells were fixed in 4% PFA for 10 min, and then I-TIP and N-TIP were incubated at 4 °C for 18 h. After washing with PBS, staining with CellMask™ green and Hoechst 33342, images were obtained from Operetta CLS (PerkinElmer, Waltham, MA, USA) using red (615–645 nm), green (460–490 nm), and blue (355–385 nm) excitation wavelengths. A 40X water lens was used for the 3D-based image, and the z-stack imaging was taken by dividing the size of -10 m to + 3 m by 1 m, and then combined using Harmony 4.9 (PerkinElmer) software.

### In vivo imaging

In vivo imaging of either LPS- or CG-induced inflammation using N-TIP was obtained as follows: PBS and inflammation inducing agents including 2% LPS solution or 1% carrageenan (CG) solution were subcutaneously administered to footpads of mice (n = 5, each group). At 10 min post-injection, a single dose of 0.4 mg/kg FSD Fluor™ 647 conjugated IgG antibody (IgG-FSD Fluor™ 647 called as I-TIP) or FSD Fluor™ 647 conjugated NMDAR1 antibody (NMDAR1-FSD Fluor™ 647 called as N-TIP) was intravenously administered followed by fluorescence imaging. In case of IVISense Cat B 680 FAST, the recommended dose (2 nM) was intravenously administered according to the manufacturer’s instructions.

The anti-inflammatory effect of dexamethasone (DEX) using N-TIP was evaluated as follows: 1% carrageenan (CG) solution were subcutaneously administered to footpads of mice (n = 5, each group). A single dose of 10 mg/kg DEX was administered intraperitoneally to the mice immediately after inflammation induction. At 10 min post-injection, 0.4 mg/kg N-TIP was intravenously administered, followed by in vivo fluorescence imaging. After image acquisition, the organs were removed for ex vivo imaging.

In vivo fluorescence images were acquired using an IVIS Spectrum CT (PerkinElmer). The scan times ranged from 1 to 2 s depending on the intensity of the emitted fluorescence signal. All in vivo FL signals were obtained with the following settings: Ex/Em for FSD Fluor™ 647 and IVISense Cat B 680 FAST, 651/667 and 675/693 nm; exposure time, 1–2 s; f/stop, 2; binning, 8; and field of view, 21.8. The image was thresholder to maximize visualization of the region of interest and minimize background fluorescence. Grayscale and fluorescence color images were superimposed using LIVINGIMAGE v 2.12 (PerkinElmer) and IGOR Image Analysis FX (Wave Metrics, Lake Oswego, OR, USA) software. Signal intensity is expressed in units of Total Radiant Efficiency [p/s]/[µW/cm^2^].

### Statistical analysis

All data are expressed as the mean ± standard deviation (SD) from at least three independent experiments, and statistical significance of the difference was determined by unpaired Student’s *t*-test using GraphPad Prism 6 (GraphPad Software, San Diego, CA). P-values less than 0.05 were considered statistically significant.

## Results

### NMDARs mediate LPS-induced M1 macrophage polarization

**Fig. 1:**
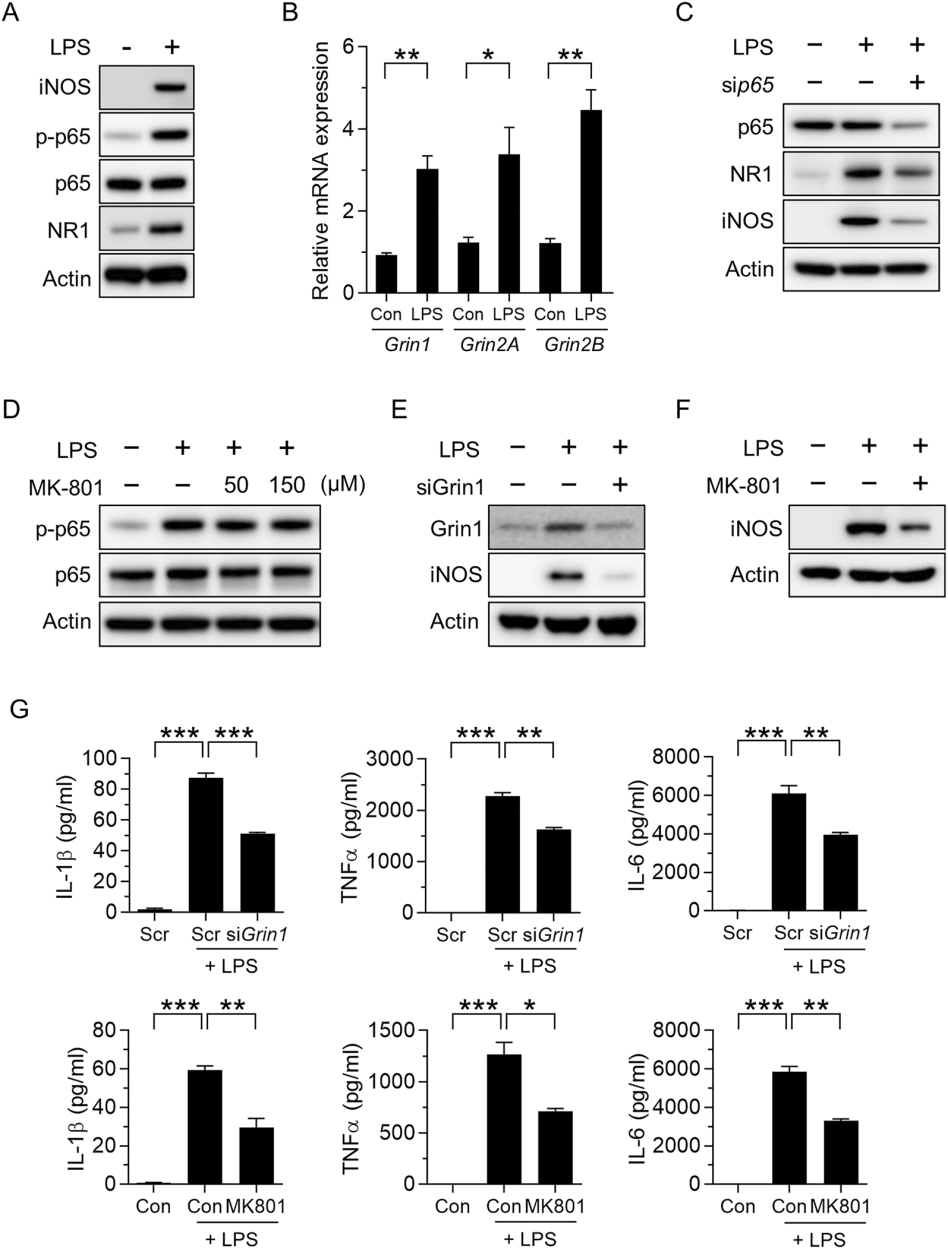
NMDAR is required for M1 macrophage polarization. **A** Levels of iNOS, phosphorylated p65, and NR1 in LPS-treated BMDMs for 24 h. **B** Relative *Grin1*, *Grin2A*, and *Grin2B* mRNA expression in LPS-treated BMDMs for 24 h. **C** Effects of LPS on the levels of p65, NR1, and iNOS in *p65*-silenced BMDMs. **D** Effects of MK-801 on the level of phosphorylated p65 in BMDMs treated with LPS for 24 h. **E**–**G** Effects of *Grin1*-targeting siRNA or MK-801 on iNOS (E and F) and pro-inflammatory cytokine secretion (**G**) in LPS-treated BMDMs for 24 h. Data are expressed as the mean ± SD of three independent experiments. *p < 0.05, **p < 0.01, ***p < 0.0005

To investigate whether NMDARs are involved in M1 polarization, we measured the NMDAR subunit level in LPS-primed primary macrophages (BMDMs). Upon LPS stimulation, macrophages polarized to the M1 state characterized by upregulation of the M1-like phenotype marker inducible nitric oxide synthase (iNOS) and NF-κB signaling, key regulators of the M1 program (Fig. 1A) [16]. The levels of NR1 protein were higher in LPS-stimulated macrophages than in control cells (Fig. 1A). We found that mRNAs of genes encoding NR1, NR2A, and NR2B were upregulated in macrophages in response to LPS stimulation (Fig. 1B). We chose to focus on NR1 subunits because NR1 is an obligatory subunit responsible for forming functional NMDARs [10, 17]. Inhibition of NF-κB with siRNA against p65 prevented LPS-induced upregulation of NR1 (Fig. 1C), which is consistent with a previous finding that NF-κB binds to the GRIN1 sequence and stabilizes it [18]. In contrast to the finding of a previous study, that is, blocking of nuclear translocation of NF-κB by several NMDA antagonists [19], pharmacological inhibition of NMDAR with MK801 did not reduce NF-κB signaling (Fig. 1D). siRNA-mediated inhibition of Grin1 or pharmacological inhibition of NMDAR with MK-801 also led to failure of LPS-induced upregulation of iNOS (Fig. 1E, F). Furthermore, the production capacity for cytokines, including IL-1β, TNF-α, and IL-6, was attenuated by silencing Grin1 and antagonizing NMDAR with MK-801 (Fig. 1G). Taken together, these results suggested that NMDARs mediate LPS-induced M1 macrophage polarization.

### NMDAR-mediated glycolysis is required for M1 macrophage polarization

**Fig. 2:**
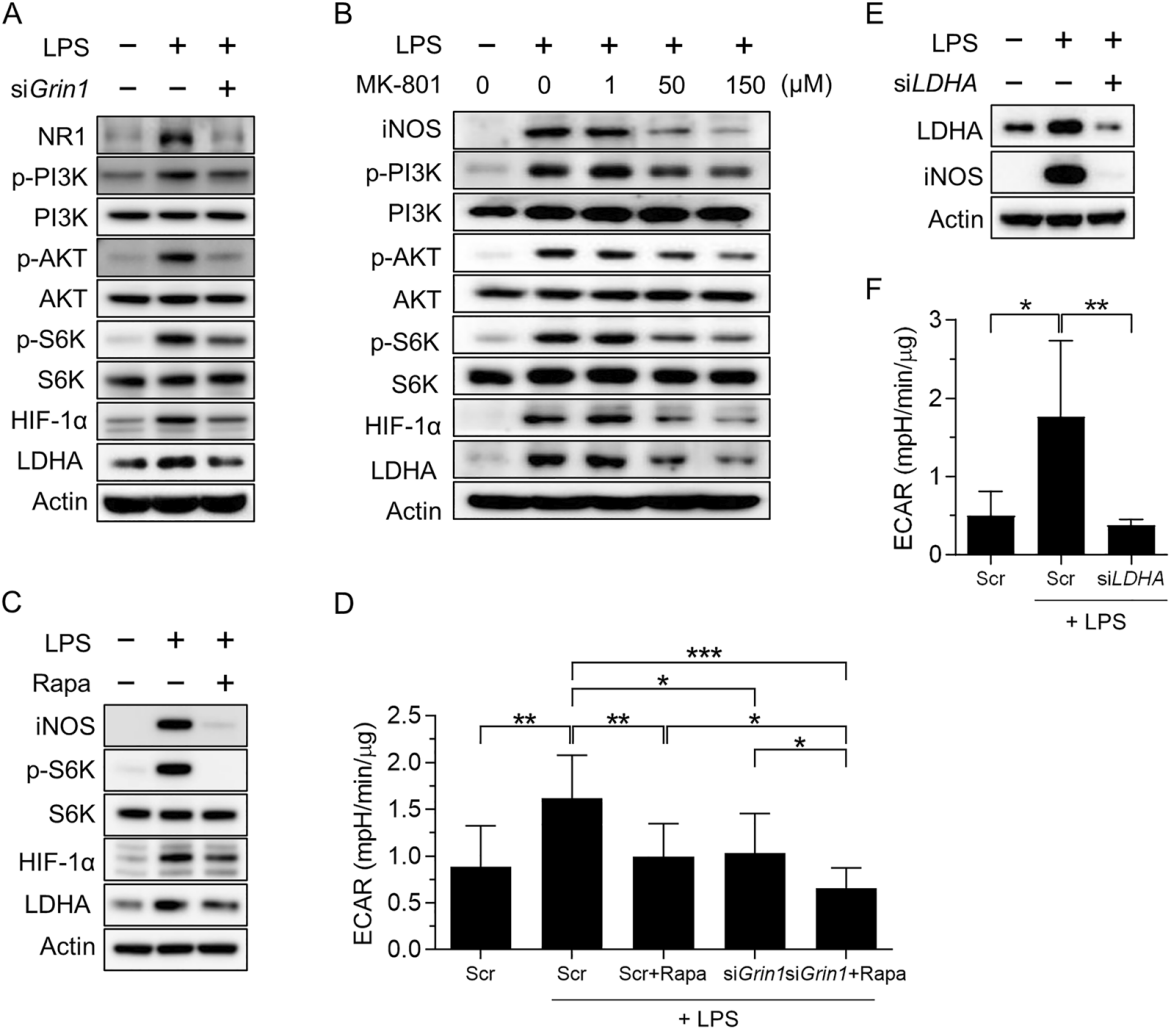
M1 polarization of macrophages requires NMDAR-induced glycolysis. **A** and **B** Effects of *Grin1*-targeting siRNA (A) or MK-801 (1, 50, 150 μM; B) on phosphorylated PI3K, AKT, and p70S6K and HIF-1α and LDHA in LPS-treated BMDMs for 24 h. **C** Effects of rapamycin on the levels of iNOS, phosphorylated S6K, HIF-1α, and LDHA in LPS-treated BMDMs for 24 h. **D** Extracellular acidification rate (ECAR) in *Grin1*-silenced or/with Rapa (rapamycin)-treated BMDMs with LPS for 24 h. **E**, **F** Effects of *LDHA*-targeting siRNA on the levels of iNOS (**E**) and ECAR (**F**) in LPS-treated BMDMs for 24 h. Data are expressed as the mean ± SD of three independent experiments.. *p < 0.05, **p < 0.01, ***p < 0.005

To investigate the mechanism by which NMDARs mediate LPS-induced M1 macrophage polarization, we investigated the PI3K/AKT/mTORC1 signaling pathway and glycolysis in LPS-treated BMDMs. As shown in Fig. 2A, the phosphorylation of PI3K (Y458), AKT (T308), and S6K (T389) was increased in response to LPS stimulation. Furthermore, levels of HIF-1α, a transcription factor implicated in M1 activation, and LDHA, an enzyme required in the last step of glycolysis, were also increased in response to LPS. Inhibition of NMDAR by silencing Grin1 or treatment with MK-801 diminished LPS stimulation-induced activation of PI3K/AKT/mTORC1 signaling and glycolysis (Fig. 2A, B). mTORC1 could reinforce the glycolytic program by translating HIF-1α mRNA or stabilizing HIF-1α expression (20); thus, we investigated whether mTORC1 is responsible for the LPS-induced glycolysis. We found that the mTORC1 inhibitor rapamycin attenuated LPS-induced upregulation of iNOS, HIF-1α, and LDHA (Fig. 2C). We confirmed the effect of NMDARs or mTORC1 on LPS-stimulated glycolysis by measuring the ECAR in macrophages. As shown in Fig. 2D, rapamycin or silencing of Grin1 significantly reduced LPS-induced glycolysis, which was lower when both mTORC1 and Grin1 were inhibited, suggesting that NMDAR regulates glycolysis at least through the mTORC1 pathway. In agreement with the above findings, silencing of LDHA decreased LPS-induced upregulation of iNOS and ECAR (Fig. 2E, F). Collectively, these findings support that NMDAR-dependent glycolysis is a critical metabolic pathway involved in M1 polarization.

### NMDAR-mediated Ca^2+^ accumulation results in LPS-stimulated glycolysis in macrophages

**Fig. 3:**
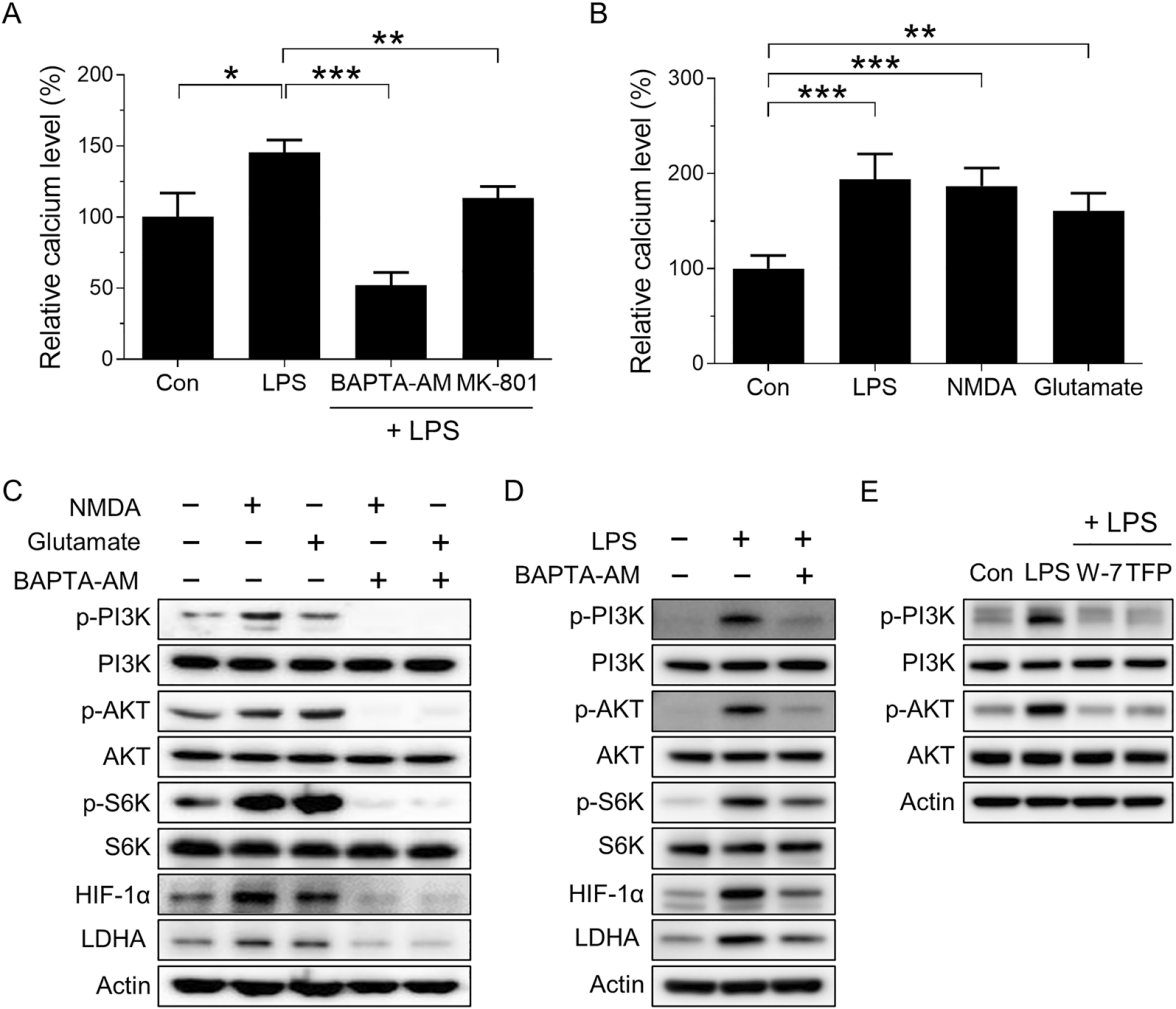
NMDAR increases glycolysis through intracellular calcium flux. **A** Effects of LPS on cytosolic Ca^2+^ level in BMDMs treated with BAPTA-AM or MK-801 for 24 h. **B** Effects of LPS, NMDA, or glutamate on cytosolic Ca^2+^ level in BMDMs. The cells were treated for 24 h. **C** Effects of NMDA, glutamate, or BAPTA-AM on the levels of the indicated proteins in BMDMs for 24 h. **D** Effects of BAPTA-AM on the levels of the indicated proteins in LPS-treated BMDMs for 24 h. **E** Effects of the calmodulin inhibitors W-7 and TFP on phosphorylated PI3K and AKT in LPS-treated BMDMs for 24 h. Data are expressed as the mean ± SD of three independent experiments. *p < 0.05, **p < 0.01, ***p < 0.005

As calcium signaling accounts for most of the NMDAR-mediated functions [9], we hypothesized that NMDAR activation-induced overload in intracellular Ca^2+^ and the consequent intracellular Ca^2+^ increase could result in activation of PI3K/AKT/mTORC1 signaling [21]. As expected, the intracellular Ca^2+^ concentration increased in LPS-stimulated BMDMs, and then returned to the baseline levels following treatment with the calcium chelator BAPTA-AM or MK-801 (Fig. 3A). Treatment with the major NMDAR agonist NMDA or glutamate increased the intracellular Ca^2+^ concentrations (Fig. 3B). Moreover, treatment of BMDM with NMDA or glutamate increased the phosphorylation of PI3K (Y458), AKT (T308), and S6K (T389), as well as the protein levels of HIF-1α and LDHA; however, this increase was attenuated by treatment with BAPTA-AM (Fig. 3C). Finally, BAPTA-AM also inhibited LPS-induced PI3K/AKT/mTORC1 signaling and glycolysis in macrophages (Fig. 3D). To further verify the mechanism by which Ca^2+^ activates PI3K signaling and its downstream components in macrophages, we analyzed the effects of calmodulin (CaM) inhibitor on this signaling in LPS-treated macrophages. Consistent with previous study results, that is, the phosphorylation of PI3K can be activated by increased intracellular Ca^2+^ level through CaM [22, 23], we also found that the CaM inhibitors W-7 and TFP suppressed LPS-induced phosphorylation of PI3K (Y458) and activity of PI3K, as evidenced by decreased phosphorylated PIP3-dependent Ser/Thr kinase AKT (T308), supporting Ca^2+^-mediated PI3K signaling (Fig. 3E). Taken together, our results demonstrated that NMDAR-mediated Ca^2+^ accumulation results in activation of PI3K/AKT/mTORC1 signaling axis and glycolysis.

### Development of NMDAR-targeting probes for visualization of M1-polarized macrophages

**Fig. 4:**
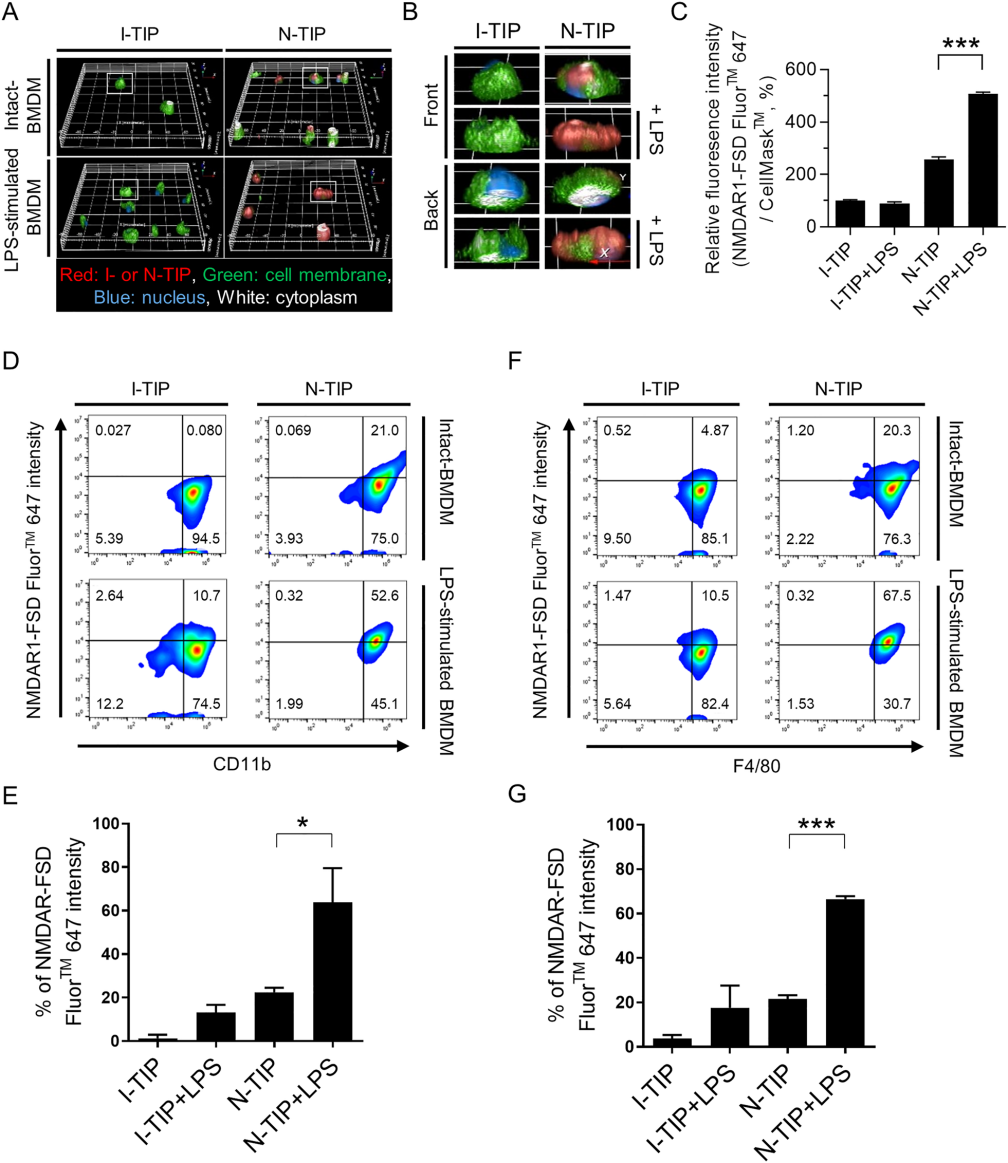
Binding of NMDAR1-FSD Fluor™ 647 (N-TIP) to LPS-stimulated BMDMs. **A** BMDMs were treated with I-TIP or N-TIP (red), CellMask™ green (green), and Hoechst 33,342 (blue) for 3D-based imaging. **B** The white box in (A) is enlarged and the front and back images are shown. **C** The intensity of the 3D-based image was determined as the total NMDAR-FSD Fluor™ 647 area compared to the total CellMask™ green area. **D**–**G** Intact-BMDMs and LPS-stimulated BMDMs were incubated with either I-TIP or N-TIP, followed by FACS analysis. Flow cytometry histogram and bar graph to demonstrate the percentage of NMDAR on CD11b^+^ (D and E) or F4/80^+^ (F and G) macrophages. Length of X- and Y-axis: 160 μm, Z-axis: 14 μm. Data are expressed as the mean ± SD of three independent experiments. *p < 0.05, ***p < 0.005

Based on the biological relevance of NMDARs in M1 macrophage polarization, we postulated that NMDARs can be significant target for visualization of the dynamics of M1 macrophage distribution in inflammatory diseases. Successful conjugation of FSD Fluor™ 647 dye to both isotype and NMDAR1 antibody was confirmed using thin layer chromatography (TLC, Additional file 2: Fig. S2), absorbance/fluorescence spectra (Additional file 2: Fig. S2), and F/P molar ratio (fluorophore to protein ratio) calculation; vide infra. Isotype-FSD Fluor™ 647 and NMDAR1-FSD Fluor™ 647 were designated as I-TIP and N-TIP, respectively (Additional file 2: Fig. S2). The F/P ratio of I-TIP was determined as 9.45, and that of N-TIP was 8.89 (See the formula described in the Methods). A 3D-based imaging technique that enables the visualization of cell surface binding of imaging probe was conducted to determine the functional binding of N-TIP on the cell membrane of LPS-stimulated BMDMs (Fig. 4A). Using this 3D-based imaging approach, we could clearly identify the cell membrane (green), nucleus (blue), cytoplasm (white), and specific binding lesion of I-TIP or N-TIP (red) in tested cells. As shown in Fig. 4A-C, red signals were not detectable in intact-BMDMs and LPS-stimulated BMDMs when I-TIP was applied. However, N-TIP binding lesion (red signal) was observed in both intact-BMDMs and LPS-stimulated BMDMs. Importantly, red signals were considerably higher in LPS-stimulated BMDMs than in intact-BMDMs (Additional file 3: Fig. S3A and Additional files 6, 7, 8 and 9: Supporting Movie 1–4). To further confirm the findings of the 3D-based imaging approach, intact-BMDMs and LPS-stimulated BMDMs were incubated with N-TIP (or I-TIP) and M1 macrophage-specific antibodies, such as CD11b and F4/80 Ab, followed by FACS analysis. The results revealed that N-TIP but not I-TIP enabled the detection of basal expression of NMDAR1 in intact-BMDMs (Fig. 4D–G), consistent with the in vitro findings from both western blot analysis and 3D-based cell imaging (Fig. 1 and Fig. 4A-C). Importantly, treatment with LPS increased NMDAR1 expression on CD11b^+^ BMMDs (Fig. 4D, E). Similarly, significant upregulation of NMDAR1 expression was observed in F4/80^+^ BMDMs stimulated with LPS (Fig. 4F, G). Consistent with these findings, intensive FL signals were detectable in LPS-stimulated BMDMs but not in intact-BMDMs (Additional file 3: Fig. S3B).

### N-TIP-mediated bio-imaging in inflammatory lesions.

**Fig. 5:**
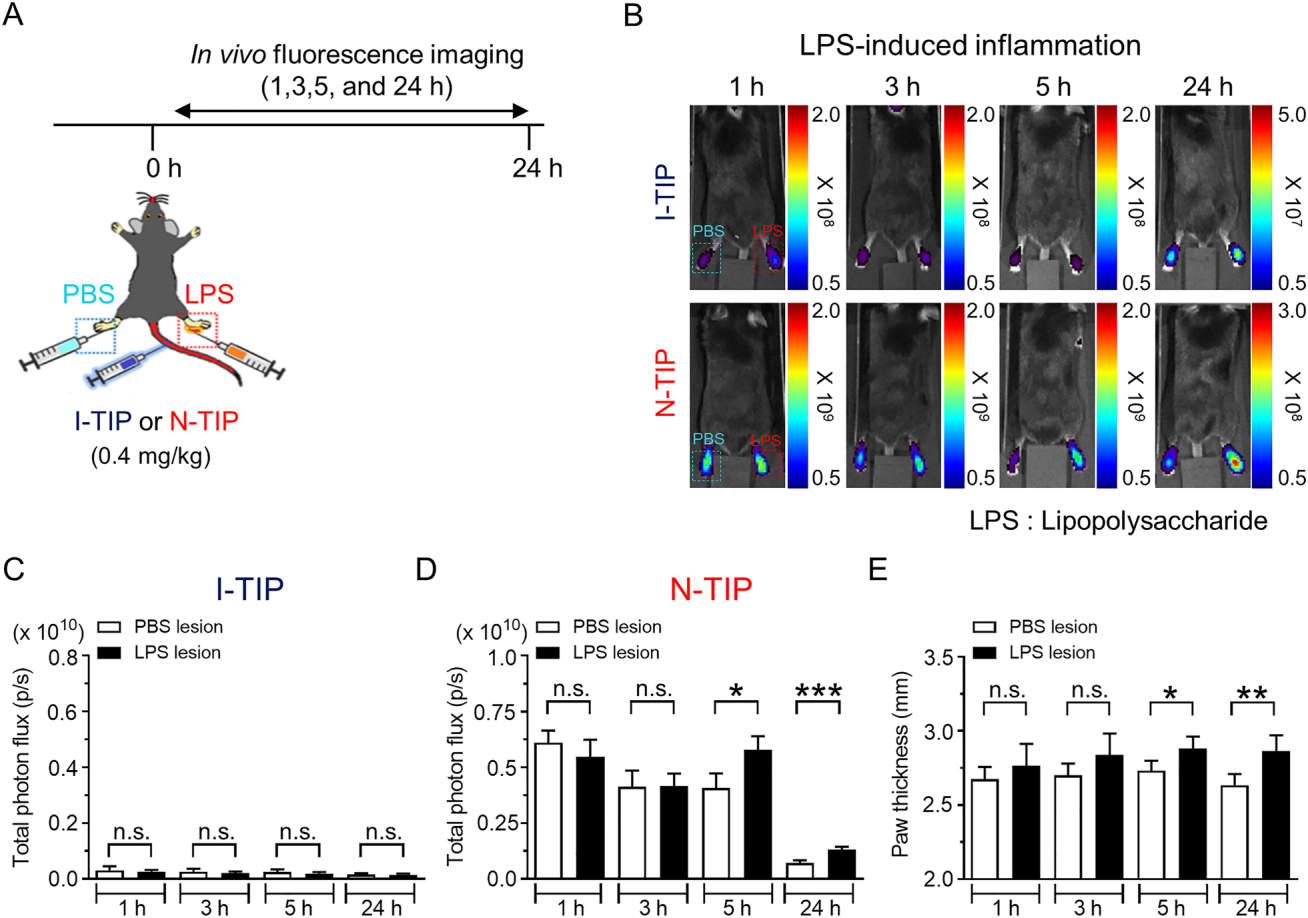
In vivo visualization of LPS-induced inflammation in living mice using N-TIP. **A** Schematic procedures for in vivo study. For inflammation induction, LPS solution was injected into the paw of mice, immediately followed by a 0.4 mg/kg N-TIP injection. In vivo fluorescence imaging was conducted at indicated times. **B** In vivo detection of LPS-induced inflammatory lesion using N-TIP. **C**, **D** Quantification of FL signals in (B) panel. **E** Measurement of paw thickness after PBS or LPS injection. Data are the mean ± SD of 5 mice. *p < 0.05, **p < 0.01, ***p < 0.0005, n.s., not significant

**Fig. 6:**
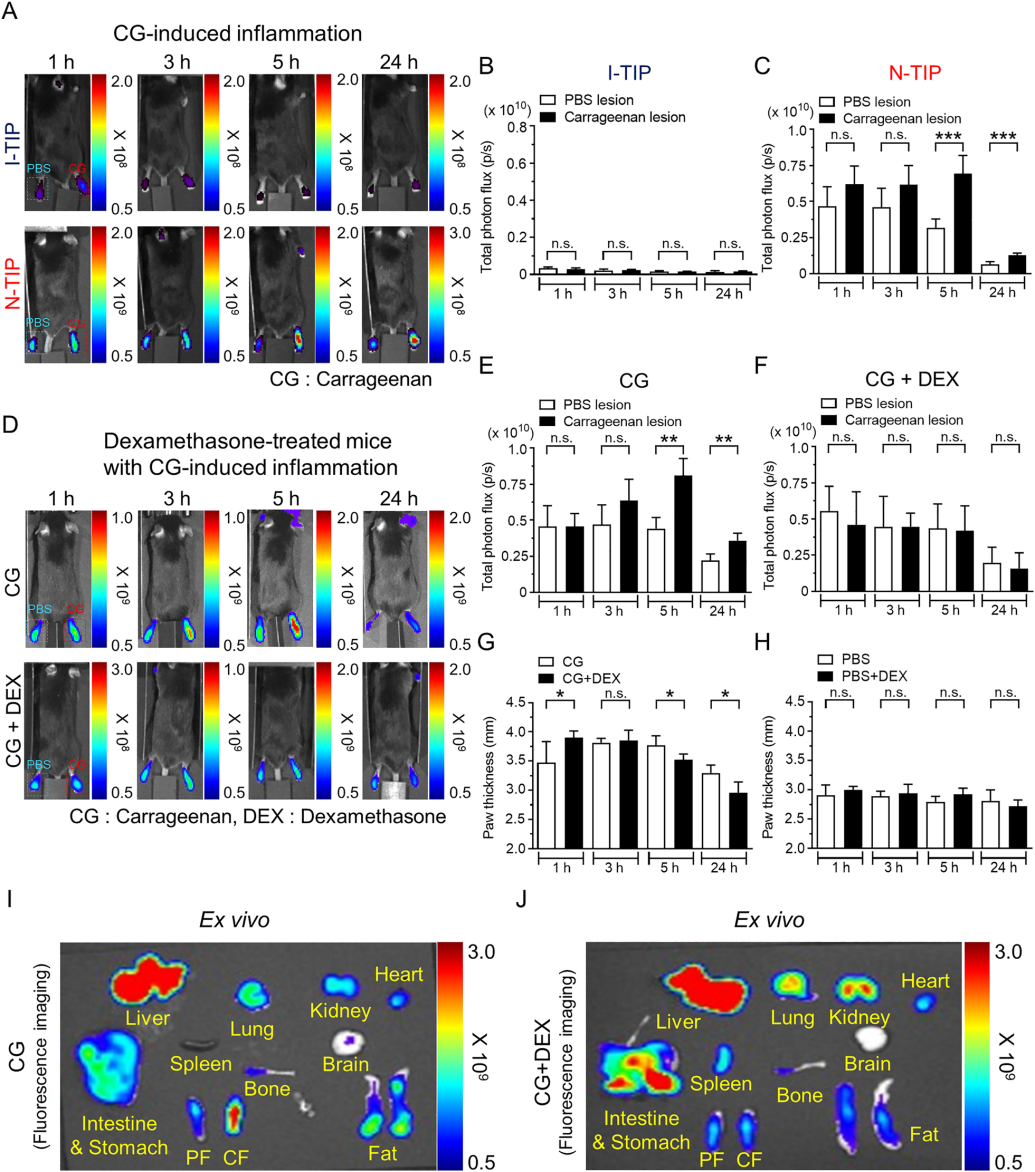
In vivo visualization of CG-induced inflammation and evaluation of the therapeutic efficacy of anti-inflammatory agents in living mice using N-TIP. **A** Representative fluorescence image showing CG-induced inflammatory lesion using N-TIP. **B**, **C** Quantification of FL signals in (A) panel. **D** In vivo fluorescence images showing the anti-inflammatory effects of DEX. Upon inflammation induction, DEX was administered, and then 0.4 mg/kg N-TIP was injected via the tail vein. In vivo imaging was performed at indicated times. **E**, **F** Quantification of FLs signals in (D) panel. (G and H) Measurement of paw thickness by DEX after CG (G) or PBS (H) injection. **I**, **J** Ex vivo imaging of excised organs from CG- (I) or CG + DEX- (J) injected mice. Mice were sacrificed at 5 h post-inflammation induction, and the respective organs were excised, followed by ex vivo imaging. Data are the mean ± SD of 5 mice. *p < 0.05, **p < 0.01, ***p < 0.0005, *ns* not significant, *CF* carrageenan injected foot, *PF* PBS-injected foot

Based on the finding of the selective binding of N-TIP to M1-polarized macrophages, we next evaluated the feasibility of using N-TIP as a novel imaging agent for inflammatory response in living mice. For inflammation models, LPS-induced inflammation and CG-induced inflammation, which are widely used in several studies on inflammation, were adopted [24–26]. Upon inflammation induction, the mice were administered 0.4 mg/kg N-TIP via intravenous injection, and in vivo fluorescence was measured at indicated times (Fig. 5A). As expected, N-TIP revealed an inflamed lesion in the LPS-injected paw but not in the PBS-injected paw at 5 h post-injection of N-TIP (Fig. 5B-D). The LPS-induced inflamed lesion could be observed until 24 h. However, there was no difference in FL signals between the control paw and LPS-injected paw when I-TIP was applied. Consistently, the paw thickness was significantly increased from 5 to 24 h in the LPS-injected paws compared to that of PBS-injected paws (Fig. 5E). In the case of CG-induced inflammation, we could first detect inflamed lesions at 5 h post-inflammation using N-TIP. The CG-induced inflamed lesion could also be observed until 24 h (Fig. 6A–C). Similar to that in LPS-induced inflammation, I-TIP could not distinguish between a control and an inflamed lesion. We further conducted in vivo imaging study with IVISense Cat B 680 FAST, a well-known imaging agent for macrophage detection in inflamed lesions [27–32], and N-TIP. The inflamed lesions observed with N-TIP were comparable to those observed with IVISense Cat B 680 FAST, which suggests that NMDAR-flour 647 could be applied to track pro-inflammatory macrophages (Additional file 4: Fig. S4A, B). To exclude the biological effect of N-TIP on the NMDAR level and its related signaling, we isolated peritoneal macrophages from mice i.p. injected with N-TIP or I-TIP with LPS. We observed that N-TIP neither increased the level of NR1 and phosphorylation of PI3K, AKT, and S6K compared with I-TIP, suggesting that N-TIP could be used as a bio-imaging probe without affecting signaling pathways in mice (Additional file 5: Fig. S5).

We next attempted to determine whether the N-TIP-mediated macrophage imaging approach has a potential for evaluating the therapeutic effects of anti-inflammatory agents in living mice. DEX has been widely adopted to treat inflammatory diseases in pre-clinical and clinical settings [33]. CG-induced inflammation was established, followed by DEX treatment via intraperitoneal injection to evaluate the therapeutic efficacy of DEX. As illustrated in Fig. 6D–F, strong fluorescence signals were detected in the inflamed lesions for up to 24 h following the induction of inflammation. However, DEX treatment significantly reduced the fluorescence signals from the inflamed lesions compared with those in the control mice. Consistent with the in vivo findings, paw thickness was significantly reduced in DEX-treated mice (Fig. 6G, H). Bio-distribution of N-TIP was further confirmed. Consistent with the in vivo findings, ex vivo imaging demonstrated an intensive fluorescence signal in the CG-injected paw (Fig. 6I) and significant reduction in DEX-treated mice (Fig. 6J).

## Discussion

**Fig. 7:**
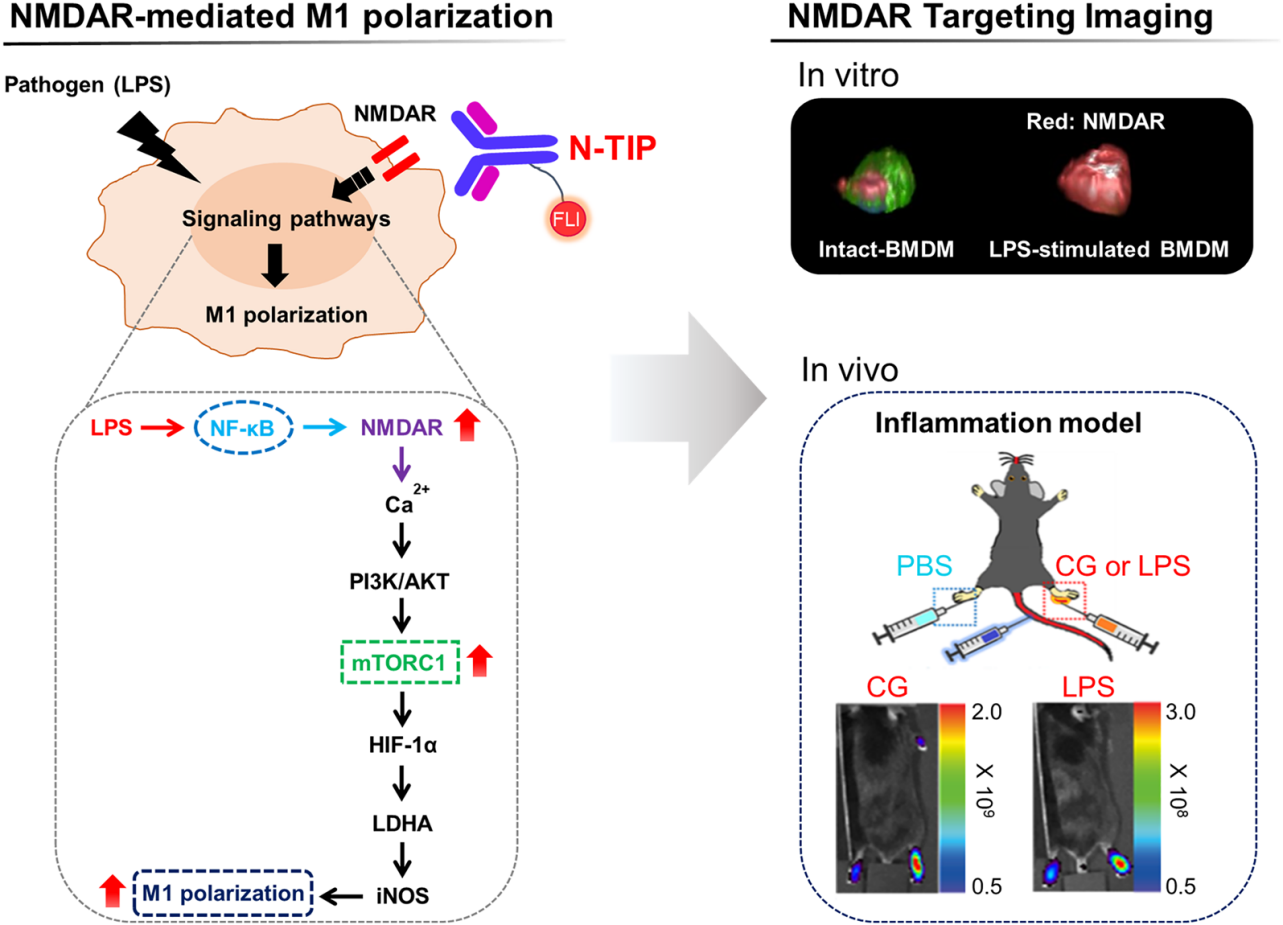
Schematic showing the mechanism by which NMDARs induce M1 macrophage polarization and the development of NMDAR-targeting imaging probe for evaluation of macrophage-mediated inflammation

The data presented herein revealed the biological role of NMDARs in LPS-stimulated macrophages and identified the mechanisms by which NMDARs mediate metabolic reprogramming to ensure M1 polarization. Furthermore, we successfully demonstrated the feasibility of NMDAR-targeting imaging to visualize inflammatory lesions of mice using an optical imaging system (Fig. 7).

NMDARs are mainly associated with neurological diseases; however, accumulating evidence shows that they also participate in various inflammatory disorders, such as pulmonary fibrosis and atherosclerosis as well as wound healing [34–36]. Recently, the pro-inflammatory role of NMDARs in immune cells has been proposed [37, 38]. In line with these studies, our data showed that LPS-stimulated macrophages exhibit the phenotype of M1 polarization through activation of NMDARs. Pro-inflammatory M1 macrophages rely mainly on glycolysis and exhibit impairment of mitochondrial oxidative phosphorylation [37]. Studies show that the AKT/mTORC1 pathway potentiates the glycolytic program by translating HIF-1α mRNA or by stabilizing HIF-1α expression which, in turn, activates the transcription of glycolytic genes, including PDK1 and LDHA [39–42].

Although NMDAR activation is well known to increase intracellular Ca^2+^, which acts as an important intracellular signal messenger for PI3K/AKT activation, it is largely unknown whether NMDAR activation is implicated in the glycolysis required for M1 polarization. The present study demonstrated that LPS stimulated NMDAR-mediated Ca^2+^ influx, which increased PI3K/AKT/mTORC1 signaling and glycolysis, subsequently facilitating M1 macrophage polarization. Importantly, NF-κB-induced increase in the level of NR1 is required for PI3K/AKT/mTORC1 signaling in macrophages. Given that the stability, trafficking, and expression of NMDARs are known to be dynamically regulated by post-translational modifications such as phosphorylation, ubiquitination, and palmitoylation [43, 44], more comprehensive experiments to elucidate the dynamic regulation of NMDAR are required. Taken together, these findings indicated the possibility of using NMDARs as a target to track pro-inflammatory macrophages in inflammatory diseases.

Among the various imaging technologies, optical imaging with fluorescence dyes is a useful tool for monitoring diverse cellular activities and migration of macrophages in inflammatory lesions due to the high spatial and temporal resolution, deep tissue penetration, and low tissue autofluorescence of fluorescent imaging [45–47]. Thus, to explore the possibility of NMDAR-mediated macrophage imaging, fluorescence bio-imaging was performed using an NMDAR-selective antibody, which enabled non-invasive, sensitive, and quantitative monitoring of macrophage infiltration into inflamed lesions. For the infrared imaging probe, FSD Fluor™ 647 (Ex/Em, 651/667 nm) was adopted because it has an excellent sensitivity, is biocompatible, and is easy to label for antibody conjugation. Before in vivo imaging study, we tested whether N-TIP can selectively react with LPS-stimulated BMDMs using 3D-based imaging examination and FACS analysis. The results of both 3D-based imaging technique and FACS analysis clearly revealed the specific binding of N-TIP to LPS-stimulated BMDMs, indicating that N-TIP exhibits selective binding to M1-polarized macrophages, which will guarantee in vivo imaging application. Furthermore, in vivo imaging approach with N-TIP showed inflamed lesions in two different inflammation models, which was consistent with the results of paw thickness, suggesting that the N-TIP-mediated imaging approach is a feasible method for monitoring M1 macrophages in vivo. If macrophage imaging with N-TIP can be applied to future clinical situations, it should be useful to evaluate anti-inflammation agents in living organisms. Indeed, the present study showed that the N-TIP-mediated macrophage imaging technique allows for the evaluation of the therapeutic effects of DEX in mice with CG-induced inflammation, which was well correlated with the results of paw thickness measurement. Taken together, these findings suggest that NMDAR-mediated macrophage imaging is a feasible approach for evaluating macrophage-mediated inflammation in living subjects.

## Conclusion

The present study demonstrated that NMDAR-mediated glycolysis plays a critical role in M1 macrophage-related inflammation. Moreover, our results suggest that NMDARs can be used to monitor dynamics behaviors of M1 macrophages and that our NMDAR-mediated imaging technique may be useful in research on inflammatory response in vivo.

## Supplementary Information

**Additional file 1: Figure. S1.** TLC plate comparison of I-TIP (left) and N-TIP (right). (SiO_2_; 2-propanol 2.0 eq: n-propanol 4.0 eq: ethyl acetate 1.0 eq: water 3.0 eq), left spot, FSD Fluor^TM^ 647 dye, middle spot, Unpurified FSD Fluor^TM^ 647-Antibody conjugate, right spot, Purified FSD Fluor^TM^ 647-Antibody conjugate.

**Additional file 2: Figure. S2**. Absorbance/Fluorescence spectra of I-TIP (left) and N-TIP (right).

**Additional file 3: Figure. S3.** (A) *In vitro* visualization of N-TIP binding in LPS-stimulated BMDMs using 3D-based imaging technique. BMDMs were treated with I-TIP or N-TIP (red), CellMask^TM^ green (green), and Hoechst 33342 (blue) for 3D-based imaging. Length of X- and Y-axis: 300 μm, Z-axis: 14 μm. (B) *In vitro* fluorescent imaging of tubes containing intact BMDMs and LPS-stimulated BMDMs labeled with N-TIP.

**Additional file 4: Figure. S4.** *In vivo* visualization of CG-induced inflammation in live mice using N-TIP and IVISense Cat B 680. (A) A representative fluorescence image showing CG-induced inflammatory lesions using N-TIP and IVISense Cat B 680. For inflammation induction, CG solution was injected into the paw of mice, immediately followed by intravenous injection of N-TIP or IVISense Cat B 680. (B) Quantification of FL signals in panel (A). Data are presented as mean ± SD of 5 mice. **p<0.01, ***p<0.0005, n.s., not significant.

**Additional file 5: Figure. S5.** Effects of I-TIP and N-TIP on NR1 and phosphorylated PI3K, AKT, and S6K in peritoneal macrophages isolated from LPS with I-TIP or N-TIP-treated mice for 24 h.

**Additional file 6: Movie S1.** Representative animation showing I-TIP binding to lesion on the cell surface of intact-BMDMs using 3D-based imaging technique.

**Additional file 7: Movie S2.** Representative animation showing N-TIP binding to lesion on the cell surface of intact-BMDMs using 3D-based imaging technique.

**Additional file 8: Movie S3.** Representative animation showing I-TIP binding to lesion on the cell surface of LPS-stimulated BMDMs using 3D-based imaging technique.

**Additional file 9: Movie S4.** Representative animation showing N-TIP binding to lesion on the cell surface of LPS-stimulated BMDMs using 3D-based imaging technique.

## Data Availability

The data supporting the conclusions of this article have been provided in this article and its additional files. In addition, all data from this study can be obtained from the corresponding author upon reasonable request.

## Abbreviations

NMDARs: *N*-Methyl-d-aspartate receptors
BMDMs: Bone marrow-derived macrophages
LPS: Lipopolysaccharide
PI3K: Phosphoinositide 3-kinase
mTORC1: Mechanistic target of rapamycin complex 1
FL: Fluorescence
ECAR: Extracellular acidification rate
iNOS: Inducible nitric oxide synthase
DEX: Dexamethasone
