# Supplementary figures and images for: N-methyl-d-aspartate receptors induce M1 polarization of macrophages: Feasibility of targeted imaging in inflammatory response in vivo
Source: Cell Biosci. 2023 Mar 30;13:69. doi: 10.1186/s13578-023-01007-5 (PMC10064586; doi:10.1186/s13578-023-01007-5)

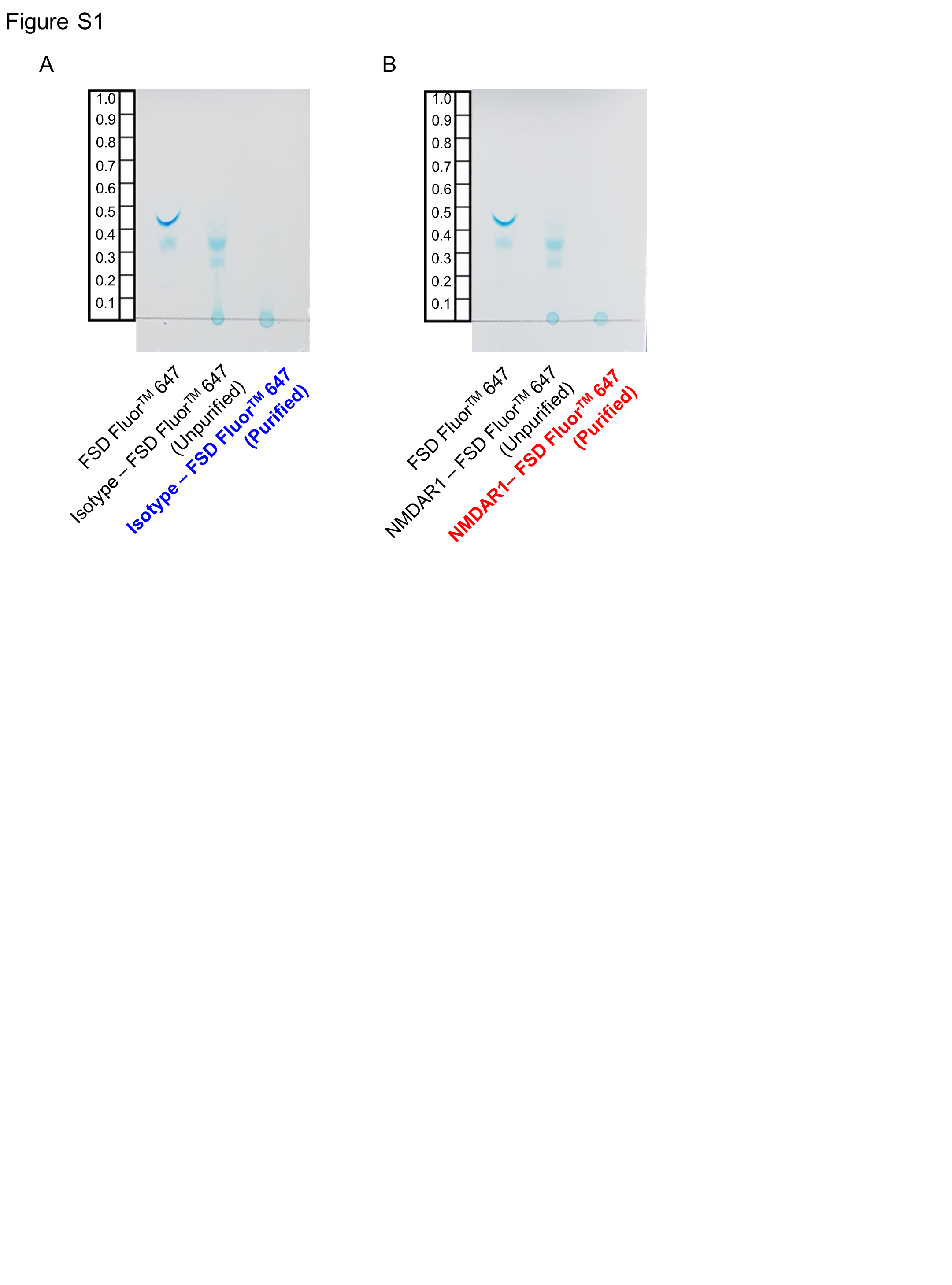

Supplement: Supplementary file 1 — Additional file 1: Figure. S1. TLC plate comparison of I-TIP (left) and N-TIP (right). (SiO2; 2-propanol 2.0 eq: n-propanol 4.0 eq: ethyl acetate 1.0 eq: water 3.0 eq), left spot, FSD FluorTM 647 dye, middle spot, Unpurified FSD FluorTM 647-Antibody conjugate, right spot, Purified FSD FluorTM 647-Antibody conjugate. [file 13578_2023_1007_MOESM1_ESM.tif]

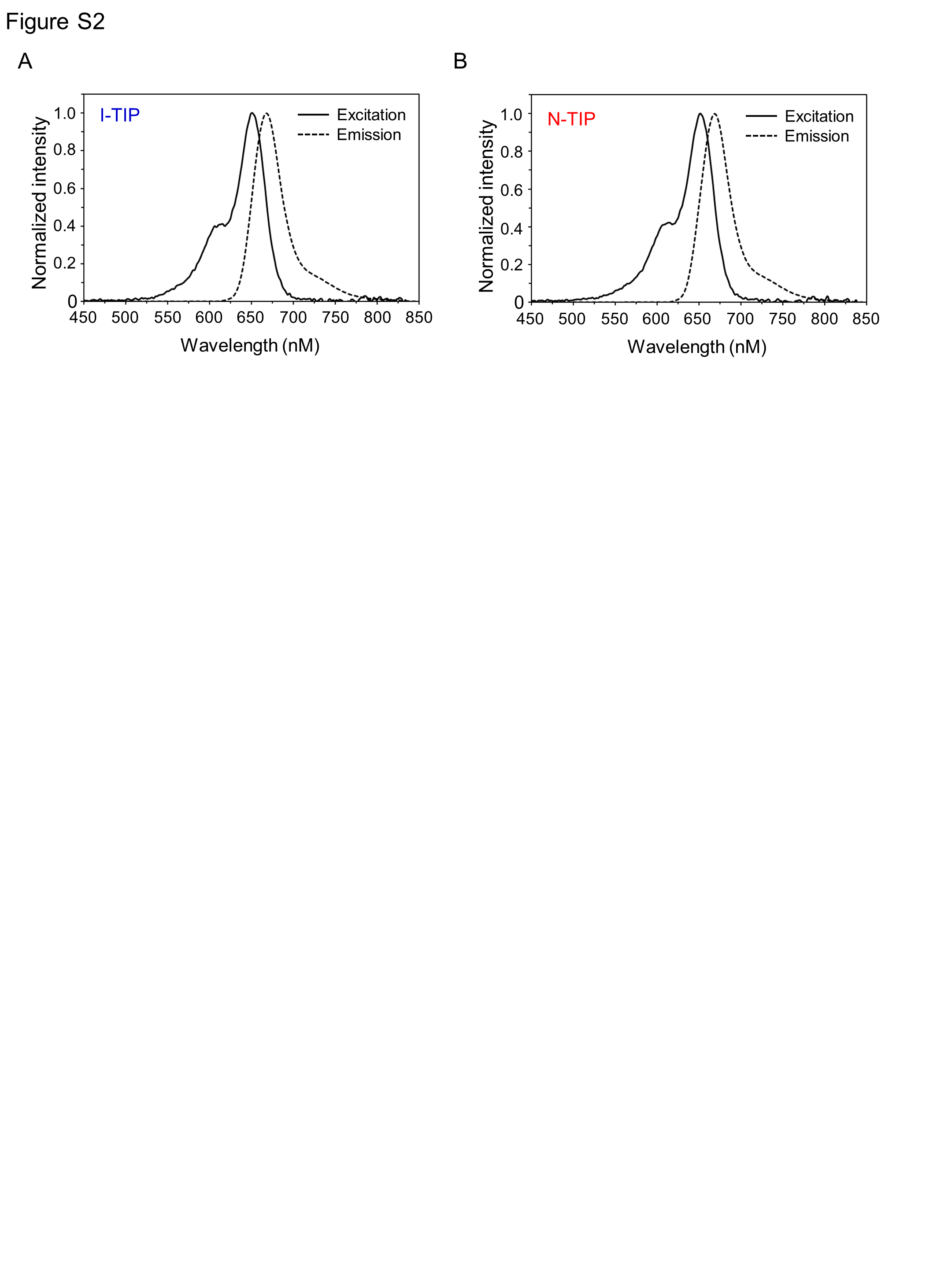

Supplement: Supplementary file 2 — Additional file 2: Figure. S2. Absorbance/Fluorescence spectra of I-TIP (left) and N-TIP (right). [file 13578_2023_1007_MOESM2_ESM.tif]

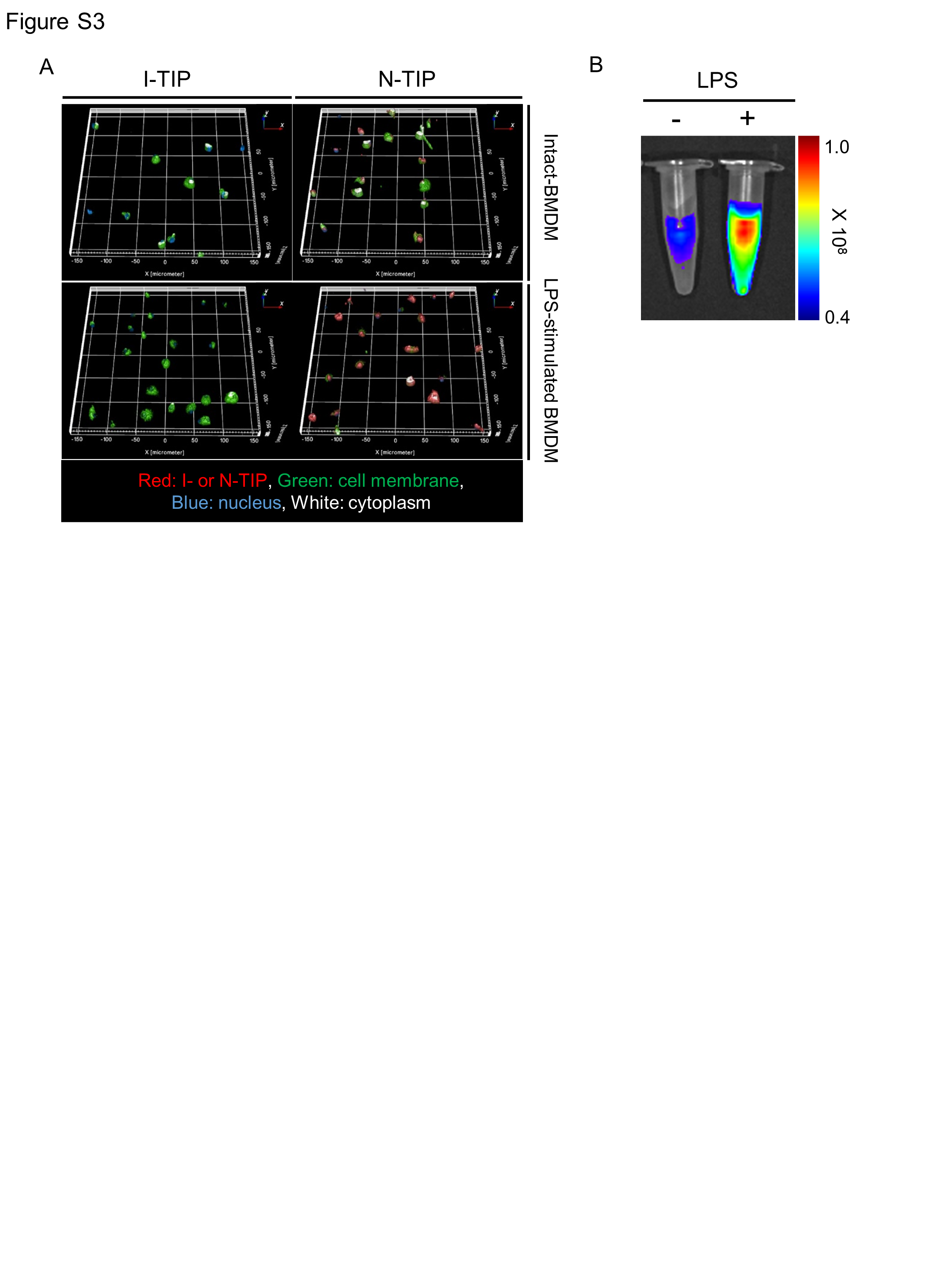

Supplement: Supplementary file 3 — Additional file 3: Figure. S3. (A) In vitro visualization of N-TIP binding in LPS-stimulated BMDMs using 3D-based imaging technique. BMDMs were treated with I-TIP or N-TIP (red), CellMaskTM green (green), and Hoechst 33342 (blue) for 3D-based imaging. Length of X- and Y-axis: 300 μm, Z-axis: 14 μm. (B) In vitro fluorescent imaging of tubes containing intact BMDMs and LPS-stimulated BMDMs labeled with N-TIP. [file 13578_2023_1007_MOESM3_ESM.tif]

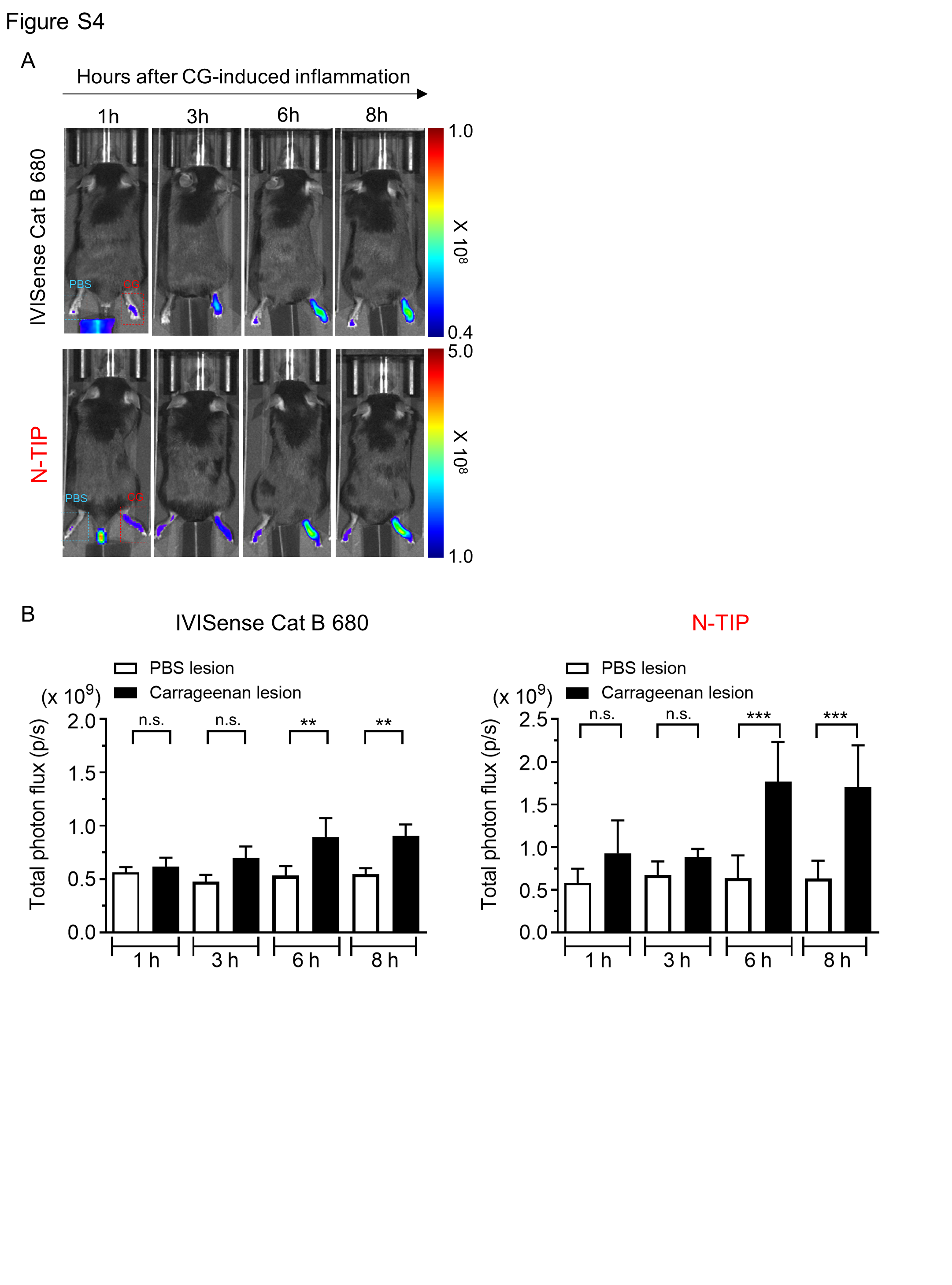

Supplement: Supplementary file 4 — Additional file 4: Figure. S4. In vivo visualization of CG-induced inflammation in live mice using N-TIP and IVISense Cat B 680. (A) A representative fluorescence image showing CG-induced inflammatory lesions using N-TIP and IVISense Cat B 680. For inflammation induction, CG solution was injected into the paw of mice, immediately followed by intravenous injection of N-TIP or IVISense Cat B 680. (B) Quantification of FL signals in panel (A). Data are presented as mean ± SD of 5 mice. **p<0.01, ***p<0.0005, n.s., not significant. [file 13578_2023_1007_MOESM4_ESM.tif]

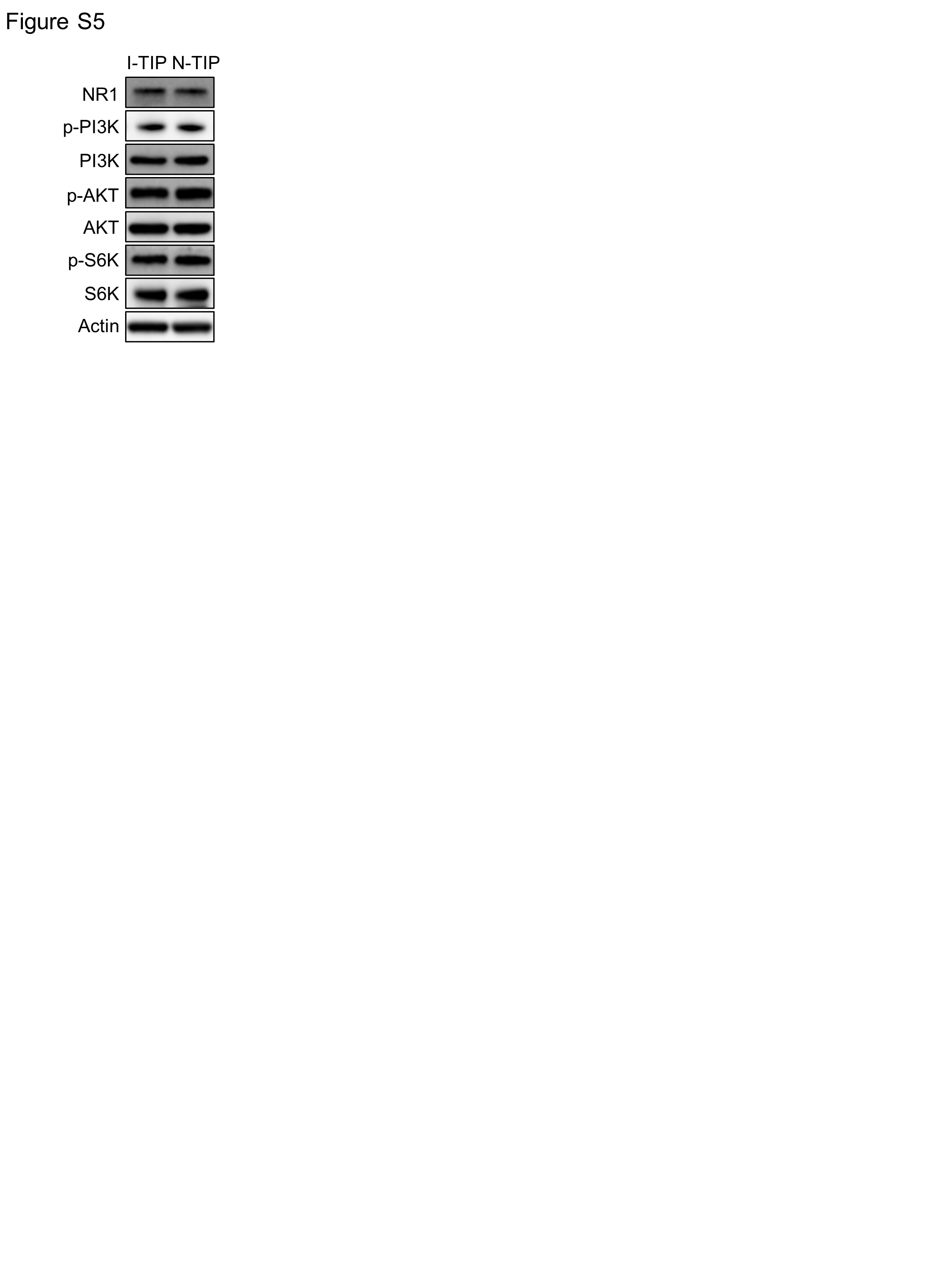

Supplement: Supplementary file 5 — Additional file 5: Figure. S5. Effects of I-TIP and N-TIP on NR1 and phosphorylated PI3K, AKT, and S6K in peritoneal macrophages isolated from LPS with I-TIP or N-TIP-treated mice for 24 h. [file 13578_2023_1007_MOESM5_ESM.tif]
